# Muscle tremors observed in white rhinoceroses immobilised with either etorphine–azaperone or etorphine–midazolam: An initial study

**DOI:** 10.4102/jsava.v92i0.2142

**Published:** 2021-06-28

**Authors:** Mary Nasr, Leith C.R. Meyer, Peter Buss, Maria C. Fàbregas, Robin D. Gleed, Jordyn M. Boesch, Friederike Pohlin

**Affiliations:** 1 Oradell Animal Hospital, Paramus, New Jersey, United States of America; 2 Section of Anaesthesiology and Pain Medicine, Department of Clinical Sciences, Cornell University College of Veterinary Medicine, Ithaca, New York, United States of America; 3 Centre for Veterinary Wildlife Studies, Faculty of Veterinary Sciences, University of Pretoria, Onderstepoort, South Africa; 4 Department of Paraclinical Sciences, Faculty of Veterinary Sciences, University of Pretoria, Onderstepoort, South Africa; 5 Veterinary Wildlife Services, Kruger National Park, South African National Parks, Skukuza, South Africa; 6 Department of Production Animal Studies, Faculty of Veterinary Sciences, University of Pretoria, Onderstepoort, South Africa; 7 Research Institute of Wildlife Ecology, Department of Interdisciplinary Life Sciences, University of Veterinary Medicine, Vienna, Austria

**Keywords:** white rhinoceros, muscle tremors, etorphine, midazolam, azaperone, immobilisation, muscle relaxation, butorphanol

## Abstract

Etorphine–azaperone is the most commonly used drug combination for chemical immobilisation of free-ranging white rhinoceroses, but causes several profound physiological disturbances, including muscle tremors. The addition of benzodiazepine sedatives, such as midazolam, has been proposed to reduce the muscular rigidity and tremors in immobilised rhinoceroses. Twenty-three free-ranging, sub-adult white rhinoceros bulls were darted and captured using a combination of etorphine plus either azaperone or midazolam. Skeletal muscle tremors were visually evaluated and scored by an experienced veterinarian, and tremor scores and distance run were compared between groups using the Wilcoxon rank sum test. No statistical differences were observed in tremor scores (*p* = 0.435) or distance run (*p* = 0.711) between the two groups, and no correlation between these variables was detected (*r* = –0.628; *p* = 0.807). Etorphine–midazolam was as effective as etorphine–azaperone at immobilising rhinoceroses, with animals running similar distances. Although the addition of midazolam to the etorphine did not reduce tremor scores compared to azaperone, it might have other beneficial immobilising effects in rhinoceroses, and further investigation is necessary to elucidate possible methods of reducing muscle tremoring during chemical immobilisation of rhinoceroses.

## Introduction

The near-threatened Southern white rhinoceros (*Ceratotherium simum simum*) must be managed by conservationists using strategies that necessitate chemical immobilisation (Miller & Buss 2015). Etorphine–azaperone is the most commonly used drug combination for chemical immobilisation of free-ranging white rhinoceroses (Miller & Buss 2015). However, this combination causes profound physiological disturbances including tachycardia, systemic hypertension, hypoxaemia, hypercapnia, respiratory acidaemia and muscle tremors (Miller & Buss 2015). Current research suggests that muscle tremors play a central role in the development of hypoxaemia and hypercapnia in etorphine-immobilised rhinoceroses by increasing the metabolic rate and oxygen consumption (Buss et al. 2018). Therefore, it is important to reduce the incidence and severity of muscle tremors in order to improve the safety of rhinoceros immobilisation.

The addition of benzodiazepine sedatives, such as midazolam, has been proposed to reduce muscular rigidity and tremors in immobilised rhinoceroses (De Lange et al. 2017; Pohlin et al. 2020a). The drug’s effect on gamma-aminobutyric acid (GABA_A_) receptors results in skeletal muscle relaxation, making it a viable candidate to reduce muscle tremors (Izwan et al. 2018). However, the effects of benzodiazepines on muscle tremors in white rhinoceroses have not yet been adequately studied.

The aim of this study was to determine whether muscle tremors in white rhinoceroses are less severe during etorphine–midazolam immobilisation than during standard etorphine–azaperone immobilisation. The primary hypothesis was that muscle tremors would be less severe during etorphine–midazolam compared to etorphine–azaperone immobilisation.

## Materials and methods

Data for this initial study were opportunistically collected from 23 free-ranging, sub-adult white rhinoceros bulls captured within the Kruger National Park (S24°59.696ʹE031°35.217), South Africa, for management purposes. Details concerning the immobilisation and data collected from these rhinoceroses are reported in Pohlin et al. (2020a). Briefly, rhinoceroses were darted from a helicopter with a combination of etorphine (3 mg – 4 mg, intramuscular [IM]; 9.8 mg/mL; Captivon^®^, Wildlife Pharmaceuticals) plus either azaperone (*n* = 11, IM; azaperone tartrate 50 mg/mL; Wildlife Pharmaceuticals) or midazolam (*n* = 12, IM; Dazonil^®^, 50 mg/mL; Wildlife Pharmaceuticals), both at five times the etorphine dose in milligram. Four rhinoceroses (three on one occasion) were captured per day, in the early morning from different areas, alternating between the two drug combinations.

Distance run from darting to immobilisation was measured by the helicopter’s global positioning system. Once rhinoceroses could be safely handled, they were blindfolded and placed into lateral recumbency. Skeletal muscle tremors were visually evaluated by an experienced veterinarian within 10 min of recumbency, and before the administration of butorphanol (5 mg per mg etorphine; butorphanol tartrate 50 mg/mL; Wildlife Pharmaceuticals) and oxygen (10 L/min by nasal insufflation), which is the time at which previous studies have reported maximum muscle tremor intensity (Buss et al. 2018). The following criteria were used to score the muscle tremors: (1) no visible tremor; (2) slight tremor: fine trembling of legs and feet; (3) mild tremor: fine trembling of legs, feet, shoulder and chest; (4) moderate tremor: marked trembling of legs, feet, shoulder and chest and (5) severe tremor: marked trembling of the whole body and head (Buss et al. 2018; De Lange et al. 2017).

Statistical tests were performed using GraphPad Prism (GraphPad Software, 2365 Northside Dr., Suite 560, San Diego, CA, United States). Tremor scores and distance run were compared between groups using the Wilcoxon rank sum test. A Spearman’s correlation coefficient was used to correlate tremor scores with distance run. Statistical significance was set at 0.05.

### Ethical considerations

The study was approved by the University of Pretoria Animal Ethics and Research Committee (V067-17) and South African National Parks (SANParks) AUCC (009/17).

## Results

Median (minimum, maximum) tremor score in both treatment groups was 4 (2, 5); mean ± standard deviation (s.d.) was 3.91 ± 0.94 and 3.58 ± 1.00 in the etorphine–azaperone and etorphine–midazolam groups, respectively. Distance run in the etorphine–azaperone group was 800 (500–2000) m (900 m ± 460.43 m), whereas distance run in the etorphine–midazolam group was 800 (100–2000) m (941.67 m ± 553.43 m). No differences were observed in tremor scores (*p* = 0.435) or distance run (*p* = 0.711) between the two groups, and no correlation between these variables was detected (*r* = –0.628; *p* = 0.807).

## Discussion

Sympathetic upregulation associated with the administration of etorphine and, or, the stress response to capture are thought to be involved in causing tremors (Buss et al. 2018; De Lange et al. 2017). Plasma catecholamine concentrations significantly correlate with tremor scores in immobilised rhinoceroses (De Lange et al. 2017). In humans, benzodiazepines have been shown to reduce circulating plasma catecholamine concentrations and induce muscle relaxation (Stratton & Halter 1985). Midazolam, at the doses administered in this study, did not reduce muscle tremor scores in etorphine-immobilised rhinoceroses. Accordingly, midazolam failed to demonstrate a difference in circulating plasma epinephrine concentrations in the same rhinoceroses (Pohlin et al. 2020b). The benzodiazepine acts on different receptors compared to etorphine and was, therefore, unable to directly mitigate any etorphine-induced tremors. However, this effect remains to be investigated at different time points and midazolam doses used.

Etorphine and midazolam, at the doses used in this study, were as effective as etorphine and azaperone at immobilising rhinoceroses, with animals running similar distances. Although the addition of midazolam to the etorphine did not reduce tremor scores compared to azaperone, it might have other beneficial immobilising effects in rhinoceroses, such as better maintenance of anaesthetic depth, cardiovascular stability, relaxation of thoracic muscles, anxiolysis and anterograde amnesia (Izwan et al. 2018). Further studies are required to confirm these hypotheses and elucidate the causes and consequences of tremors in immobilised white rhinoceroses. Such studies should involve multiple repetitive measures and observations over time and include vital signs, blood gases and other critical cardiopulmonary variables, in order to adequately assess efforts to mitigate tremors in etorphine-immobilised white rhinoceroses. So far, butorphanol, which partially antagonises the etorphine’s effects, remains the drug of choice in mitigating tremor-induced hypoxaemia in etorphine-immobilised rhinoceroses (Buss et al. 2018; De Lange et al. 2017).
